# Evaluating the Global Intensive Feeding Therapy (GIFT) for Children with CHARGE Syndrome: A Quasi-Experimental Study

**DOI:** 10.3390/children12030362

**Published:** 2025-03-14

**Authors:** Antonella Cerchiari, Francesca Pizza, Giorgia Biondo, Carolina Giordani, Martina De Paolis, Gessica Della Bella, Massimiliano Raponi, Marco Tofani

**Affiliations:** 1 Management and Diagnostic Innovations & Clinical Pathways Research Area, Neurorehabilitation and Adapted Physical Activity Day Hospital, Bambino Gesù Children’s Hospital, IRCCS, 00050 Palidoro, Rome, Italy; francesca.pizza@opbg.net (F.P.); giorgia.biondo@opbg.net (G.B.); carolina.giordani@opbg.net (C.G.); gessica.dellabella@opbg.net (G.D.B.); 2 School of Speech and Language Therapy, University of Tor Vergata, Campus of Tivoli, 00019 Tivoli, Italy; martinadepaolis9@gmail.com; 3 Management and Diagnostic Innovations & Clinical Pathways Research Area, Medical Directorate, Bambino Gesù Children’s Hospital, IRCCS, 00165 Rome, Italy; massimiliano.raponi@opbg.net; 4 Management and Diagnostic Innovations & Clinical Pathways Research Area, Professional Development, Continuous Education and Research Service, Bambino Gesù Children’s Hospital, IRCCS, 00165 Rome, Italy; m.tofani@unilink.it; 5 Department of Life Sciences, Health and Healthcare Professions, Università degli Studi “Link Campus University”, Via del Casale di San Pio V, 44, 00165 Rome, Italy

**Keywords:** CHARGE syndrome, dysphagia, rehabilitation, GIFT, feeding, children

## Abstract

Background: This pilot investigation aimed to evaluate the efficacy of Global Intensive Feeding Therapy (GIFT) on feeding and swallowing abilities in children with CHARGE Syndrome (CS). GIFT is a novel rehabilitation program designed to leverage the principles of neuroplasticity, intensity, individualized treatment, and ecological validity. The program comprises 15 sessions conducted over one week, with sessions delivered three times per day. Methods: GIFT was preliminarily implemented in a cohort of seven children diagnosed with CS. To assess the risk of dysphagia, the Pediatric Screening–Priority Evaluation Dysphagia (PS-PED) was administered. The effectiveness of the intervention was evaluated using three instruments: the Karaduman Chewing Performance Scale (KCPS) for chewing performance, the American Speech-Language-Hearing Association National Outcome Measurement System (ASHA NOMS) for overall feeding abilities, and the Feeding Assessment Scale (FAS) to capture parents’ perceptions. Data were collected at baseline (T0), immediately post-intervention (T1), and at a six-month follow-up (T2). The Wilcoxon signed-rank test was employed for statistical analysis, and effect sizes for specific outcomes were determined using Kendall’s W. Results: The findings indicated that children with CS were at a high risk of dysphagia as measured by the PS-PED at baseline. Statistically significant improvements in chewing performance were observed at the six-month follow-up (*p* < 0.05). Feeding abilities, as measured by the ASHA NOMS, showed significant enhancement immediately post-intervention (*p* = 0.02) and at the follow-up (*p* = 0.03). Similarly, parents reported significant improvements in their children’s feeding abilities at both post-intervention and follow-up assessments (*p* = 0.02), further corroborating the clinical benefits of the intervention. Conclusions: These preliminary results suggest that GIFT may be an effective rehabilitation program for addressing feeding and swallowing disorders in children with CS. Further studies with larger sample sizes and controlled designs are warranted to substantiate these findings and refine the intervention protocol.

## 1. Introduction

CHARGE syndrome (CS) is a rare genetic disorder that affects approximately 1 in 10,000 live births [1]. It presents with a spectrum of dysmorphic and congenital anomalies that vary among affected individuals. The acronym CHARGE highlights its most common clinical features: Coloboma, Heart defects, Atresia Choanae, Retarded growth and development, Genital hypoplasia, and Ear anomalies/deafness [1,2]. The syndrome can also include hearing loss, tracheoesophageal fistula, cleft lip and palate, and cranial nerve impairment [3].

Despite variability in clinical manifestations, diagnostic criteria have been established according to both frequency and the uniqueness of specific anomalies observed in children with CS. Major criteria refer to features with higher incidence and anomalies that are uncommon in other syndromes, while minor criteria comprise less frequent and less specific manifestations (Table 1) [2].

The wide range of possible clinical features leads to a complex and heterogeneous presentation, which often has a significant impact on the patient’s quality of life and may be associated with delays in feeding abilities and dysphagia [4]. Feeding and swallowing difficulties reportedly affect 80–90% of children with CS [5], typically arising during the first year of life. Although the term “feeding difficulties” is broad, issues in CS can range from oral-phase challenges—such as poor coordination of sucking and chewing or aversive feeding behaviors—to pharyngeal-phase deficits that may involve aspiration risks. Some children experience primarily behavioral or sensory-based feeding problems (e.g., food refusal, tactile hypersensitivity), while others exhibit anatomical or neuromuscular impairments leading to impaired airway protection and the need for more specialized intervention. These problems may stem from structural anatomical factors, oral and pharyngeal sensory–motor dysfunctions, and maladaptive behaviors, all of which can compromise safe and effective feeding [4,5]. In many cases, alternative feeding methods become necessary to safeguard growth, health, and the overall quality of life of both patients and families.

Most major and minor diagnostic criteria with a structural background contribute to feeding and swallowing difficulties (e.g., cleft palate, tracheoesophageal fistula, cardiovascular defects) [6]. However, when cranial nerve dysfunction (involving nerves VII, IX, X, and XI) impacts pharyngeal control plays a central role in these disorders, as these nerves mediate both the motor and sensory aspects of the structures responsible for feeding and swallowing [5]. Key signs of delayed feeding and dysphagia include oral hypersensitivity, poor coordination of sucking–breathing–swallowing, weak sucking, masticatory dysfunction, drooling, food refusal, vomiting, and reflux [7]. In some instances, sucking, chewing, and swallowing abilities may be absent or underdeveloped, potentially leading to negative or even traumatic mealtime experiences [2,3,4].

Although feeding and swallowing disorders in CS have been widely described [3,4,5], rehabilitative efforts have received limited attention. Interventions tend to be compensatory—such as tube feeding—to ensure adequate nutrition and hydration, thereby supporting the child’s growth. However, prolonged tube feeding can delay the acquisition of critical oral sensory and motor skills [5]. When clinical conditions improve enough to allow a transition from tube feeding to oral feeding, the lack of earlier oral experience may pose substantial challenges for the child and caregivers.

There is a notable lack of evidence on the effectiveness of feeding rehabilitation interventions for children with CS. One study by Smith and colleagues [8] examined a behavioral analytic approach in four children with the syndrome, suggesting that such strategies could offer benefits. Nevertheless, such interventions should address both the oral-phase behavioral factors and the pharyngeal-phase biomechanical or neurological constraints that may contribute to aspiration and other swallowing risks. These findings underscore the need for a broader rehabilitative approach that addresses not only physiological feeding and swallowing issues but also the overall clinical, behavioral, and emotional profile of the child and family. The objective is to achieve generalized, long-lasting improvements over time.

Given the positive outcomes reported for Global Intensive Feeding Therapy (GIFT) in other syndromes, such as Autism Spectrum Disorder and Down Syndrome [9,10], the primary aim of the present study is to adapt and evaluate the effectiveness of GIFT in a small sample of children with CS. A secondary aim is to provide a more detailed characterization of the feeding and swallowing behaviors in these children, investigating how clinical and behavioral factors specific to CS—such as delayed feeding skills, food selectivity, and major and minor diagnostic criteria—may influence outcomes.

## 2. Materials and Methods

To evaluate the effectiveness of GIFT in children with CS, a pilot study was conducted between April 2024 and November 2024 at the Bambino Gesù Children’s Hospital Feeding and Swallowing Disorders Service. The Bambino Gesù Children’s Hospital Ethics Committee (Rome, Italy) approved the study (protocol number 3329_OPBG_2024). Parents or caregivers were informed of the study’s objectives and procedures, and written informed consent was obtained prior to enrollment. Participants were also free to withdraw from the study at any time or to decide whether their data could be used, without any impact on their rehabilitation pathway.

### 2.1. Participants

Children with a genetically confirmed diagnosis of CS, aged between 0 and 14 years, were recruited from the Feeding and Swallowing Disorders Service at Bambino Gesù Children’s Hospital due to swallowing difficulties. Those who had received or were currently undergoing feeding and swallowing rehabilitation were excluded.

Before the rehabilitation program, each participant was evaluated by a multidisciplinary medical team comprising an otolaryngologist, gastroenterologist, pulmonologist, nutritionist, and physiatrist. Their aim was to identify and address any pertinent medical or clinical issues (e.g., gastroesophageal reflux, constipation, vomiting, respiratory disorders) before and during the rehabilitation process. For rehabilitation, an experienced Speech and Language Therapist (SLT) conducted detailed clinical assessments of feeding and swallowing abilities [11,12], supplemented by standardized evaluation tools (see below). Instrumental examinations—Fiberoptic Endoscopic Evaluation of Swallowing (FEES), videofluoroscopic swallowing study (VFSS) [13], and scintigraphic gastric studies—were performed as necessary, based on the child’s clinical presentation, ability to cooperate, and the need for additional diagnostic information.

### 2.2. The Global Intensive Feeding Therapy (GIFT) for CHARGE Syndrome

GIFT is an intensive rehabilitation program with specific goals tailored to each condition [9,10], but it is structured around the core principles listed below:

1. Neuroplasticity and Motor Learning: This reflects the brain’s capacity to reorganize in response to novel experiences and repeated stimuli, thus facilitating functional adaptation and recovery [14]. In the context of feeding therapy, this means that with repeated practice and exposure to therapeutic activities, the central nervous system can form and strengthen neural pathways associated with oromotor skills, swallowing, and sensory integration. Through targeted exercises and incremental challenges, children gradually learn new feeding-related behaviors, refine existing ones, and replace maladaptive patterns—ultimately optimizing their ability to manage diverse food textures, coordinate sucking, chewing, and swallowing, and cope with sensory aspects of eating.
2. Intensive Rehabilitation is grounded in the principle that high-frequency, structured practice is critical for driving meaningful functional changes in pediatric populations [15]. By providing regular and repeated therapeutic sessions—often daily or multiple times per week—children have more opportunities to reinforce emerging skills and reduce maladaptive feeding behaviors. This structured approach not only allows clinicians to closely monitor progress and adapt therapy in real time but also ensures that incremental gains in oral motor coordination, sensory processing, and swallowing efficiency are consolidated into long-term feeding habits.
3. Individualized Therapeutic Approach delivers personalized treatment through a one-to-one relationship between the SLP and the child, ensuring that the therapeutic plan addresses each child’s unique needs [16]. This dynamic model of care allows the clinician to customize techniques, set attainable yet challenging goals, and provide immediate feedback, thus optimizing the child’s rate of progress. In addition, the personalized nature of treatment fosters a supportive environment, helps reduce anxiety, and builds a positive therapeutic alliance, all of which are critical for the successful acquisition and retention of new feeding or swallowing skills.
4. Ecological Environment: Conducts interventions within familiar, everyday settings, promoting the transfer of newly acquired skills to daily life [15,16]. An ecological environment emphasizes delivering therapy in a setting that closely reflects the child’s natural routines—whether at home, in the classroom, or in other real-world contexts. Children have the opportunity to practice new skills in circumstances that mirror typical mealtime experiences. This contextual alignment helps ensure that the progress made during therapy is directly transferable to regular family meals and social situations, thereby facilitating generalization of feeding behaviors and reducing the likelihood of regression once formal therapy sessions end.
5. Family-Centered Care: it recognizes that parents and caregivers are not merely observers but active collaborators who can significantly influence a child’s progress [17]. By involving caregivers in each stage of rehabilitation—from goal-setting to daily practice—the rehabilitation professional ensures a continuous exchange of information, insights, and feedback. This inclusive approach gives families a clearer understanding of the treatment rationale, as well as the techniques and exercises used to enhance feeding and swallowing skills. Moreover, empowering caregivers to implement therapeutic strategies during everyday routines can bolster the child’s consistency and motivation, facilitating faster and more lasting improvements. Frequent communication and ongoing support help caregivers feel more confident and competent in handling potential challenges, thereby reducing stress and contributing to a more positive, nurturing environment that further reinforces skill retention.

### 2.3. GIFT Protocol Overview for CHARGE Syndrome

Global Intensive Feeding Therapy (GIFT) is a structured, time-limited program founded on the principles of neuroplasticity and intensive rehabilitation. It is adapted to the unique clinical profile of CS, wherein feeding and swallowing difficulties often arise from a combination of structural anomalies, cranial nerve dysfunction, sensory deficits, and behavioral challenges. GIFT was structured for a period of a week, three sessions of rehabilitation per day (breakfast, morning snack, and lunch) for a total of 15 GIFT sessions. Below is a summary of the key components of GIFT as implemented for children with CS.

**Stabilize and Support Proper Postural Alignment**: Children with CS often have multiple sensory and motor impairments, which can compromise postural control. Frequent hospitalizations or surgeries—common in CS—further exacerbate issues such as muscle fatigue and altered muscle tone. Within GIFT, SLT and caregivers work together to determine an optimal seating arrangement that ensures the head, neck, and pelvis are properly aligned for secure airway protection and more coordinated feeding actions [9]. This may involve specialized seating systems, supportive cushions, or individualized exercises to strengthen postural and trunk stability, thereby improving respiratory coordination and long-term oromotor performance [18].

**Systematic Desensitization and Gradual Exposure**: A hallmark of CS is limited early oral experience, often due to prolonged tube feeding (e.g., nasogastric or gastrostomy) necessitated by choanal atresia, cleft palate, or other anatomical issues. GIFT prioritizes a stepwise, controlled introduction to oral feeding, focus first on non-threatening sensory exploration (smelling, touching, or visually inspecting new foods), than incremental exposures to taste, temperature, and texture, moving from lips to oral cavity and repetition throughout the day (e.g., breakfast, snacks, lunch) to reinforce positive associations with food. By breaking down each stage of food acceptance and regularly reinforcing successes, children with CS can overcome maladaptive feeding behaviors and develop confidence in trying a wider range of food textures and flavors—an especially important progression given their frequent structural or neurosensory constraints.

**Chewing and Swallowing Abilities**: Due to cranial nerve dysfunction, children with CS often struggle to synchronize biting, tongue lateralization, and airway protection. GIFT addresses these challenges through Functional Chewing Training (FuCT) [19,20], where one hand provides jaw stabilization (sometimes using a gentle rotational movement to discourage biting reflexes), and the other hand introduces food to lateral chewing surfaces. Repeated, guided practice helps distinguish jaw from tongue movements, promoting tongue lateralization, posterior transfer of the food bolus, and more effective swallowing. Additionally, supra- and infra-hyoid muscle engagement is improved, aiding laryngeal elevation and airway protection—critical factors for children with CS who frequently have reduced protective reflexes and other airway vulnerabilities [21].

**Proper Use of Infant Devices**: In CS, prolonged reliance on devices such as pacifiers, bottles, or rigid spouts can cultivate abnormal oral motor patterns. Given the potential for craniofacial anomalies—including distinctive ear structures or clefts—these patterns may further delay oral skill development or aggravate occlusal issues. Within GIFT, the SLP can evaluate the child’s dependence on infant devices and establishes a gradual transition to open cups or alternative feeding tools based on the child’s sensory readiness and oromotor capabilities [9,10]. This transition not only curbs maladaptive sucking behaviors but also fosters more age-appropriate feeding and swallowing skills.

**Dysfunctional Behavior During Mealtimes**: Dysfunctional behaviors in children with CS may be linked to multisensory difficulties, hearing or vision impairments, or prolonged negative experiences related to medical procedures and hospital stays [22]. These factors can manifest in excessive crying, rigid food preferences, or refusal of non-preferred textures during meals. However, dysfunctional behaviors can also be categorized into two further types: those arising from multisensory or sensory-integration challenges, which can lead to overstimulation or aversion to certain textures, tastes, or smells [23]; and those stemming from clinical needs that have led to structured patterns, such as reliance on alternative feeding methods (e.g., feeding tubes) that have prevented children from developing key mealtime experiences and normal hunger–satiety cycles [24]. Children who are predominantly fed through a feeding tube often exhibit altered eating behaviors, having never developed a clear sense of hunger or satiety. This lack of internal cues can contribute to food refusal and mealtime anxiety, further perpetuating maladaptive behaviors. In such cases, GIFT integrates strategies to manage enteral feeding in a structured way. For instance, in coordination with a gastroenterologist, enteral nutrition may be administered at intervals that allow for short gaps aimed at stimulating appetite and encouraging oral intake. Another common strategy is to deliver tube feeding at night, thereby ensuring adequate nutritional intake while suspending enteral feeding during daytime meals. This approach allows children to experience a more typical meal schedule and promotes the development of structured eating routines, fostering regularity and consistent mealtime habits. By combining gradual behavioral shaping with appropriate management of enteral feeds, GIFT aims to help children with CS transition toward more active, voluntary participation in mealtime. Over time, this can reduce problematic feeding behaviors, increase oral intake, and enhance the overall feeding experience for both the child and caregivers.

**Caregiver Education and Training**: Family involvement is crucial in CS due to the complex, ongoing nature of medical care and the need for consistent follow-up [17]. Throughout GIFT, caregivers observe each therapy session, learning how the SLP guides the child’s posture, sensory exposure, and mealtime structure. In fact, hands-on practice is carried out under supervision, enabling caregivers to gain confidence and fine techniques. This family-centered approach ensures that therapeutic gains—such as expanded food repertoires or improved oromotor coordination—transfer to home routines and other community settings.

**Training in Children with Severe Dysphagia**: Many children with CHARGE syndrome (CS) have significant structural anomalies (e.g., choanal atresia, airway malformations) or a high risk of aspiration, making oral intake unsafe. Even in these cases, GIFT can provide opportunities for taste trials or small-volume practice sessions to maintain oral sensorimotor stimulation. We do not necessarily aim for full oral feeding at this stage, but rather seek to preserve an “oral dimension”—ensuring that children continue to have some form of oral experience despite severe dysphagia.

In practice, working with children who present severe dysphagia requires combining management techniques (e.g., ensuring adequate nutrition and airway safety) with rehabilitative approaches (e.g., sensory stimulation and oral motor activation). Even when a tracheal cannula is in place, GIFT includes sensory and motor exercises that help maintain oral skills. These small, carefully supervised feeding trials or oral stimulations are designed to keep the child engaged in the process of tasting and chewing, no matter how limited that engagement may be.

A decannulation protocol may also be implemented in coordination with otorhinolaryngology specialists [25,26]. This often involves gradually reducing the cannula’s diameter over time while introducing a speaking valve to encourage phonation and airflow through the upper airway. As part of the process, the SLP teaches the child to tolerate intermittent occlusion of the cannula (capping), allowing them to practice breathing independently. Once the child tolerates the cannula capped and appropriate evaluations have been performed—such as endoscopic assessments, overnight oximetry, or polysomnography to ensure stable oxygen saturation and carbon dioxide levels—the cannula can be removed. Children who successfully undergo decannulation can then follow the GIFT protocol as previously described, focusing more intensively on oral feeding skills. For those who continue to require a tracheal cannula, therapy emphasizes secretion management, oral hygiene (including how to safely aspirate secretions), and ongoing oral stimulation. Parents or caregivers receive training in these procedures, enabling them to manage their child’s airway needs and support oral motor activities at home. By adapting the protocol to the child’s capabilities at each stage—whether or not decannulation is possible—GIFT helps preserve orofacial function and lays the groundwork for future improvements in feeding and swallowing skills.

### 2.4. Assessment Tools and Outcome Measures

To assess the effectiveness of GIFT, the following instruments were administered by an experienced SLP at three time points—initial assessment (T0), after intensive treatment (T1), and at a 6-month follow-up (T2):

**American Speech-Language-Hearing Association National Outcome Measurement System (ASHA NOMS)**: This standardized instrument is designed to quantitatively capture an individual’s swallowing and feeding abilities across a seven-level scale, where each successive level represents a lower degree of dependency on external feeding supports and a higher capacity for safe oral intake [27]. At the lower end of the spectrum (Level 1), the individual is not able to swallow safely by mouth, relying entirely on non-oral methods (e.g., nasogastric tube, gastrostomy) to meet nutritional needs. At the highest level (Level 7), the individual can independently eat a variety of textures and consistencies without requiring specialized strategies or caregiver assistance. Intermediate levels reflect varying combinations of diet modifications, cueing, and compensatory techniques, providing a nuanced portrait of the person’s overall swallowing efficiency and risk of aspiration. Beyond offering a snapshot of swallowing function at a single point in time, ASHA NOMS can be used longitudinally to monitor changes. Clinicians may observe a child progressing from total reliance on tube feeding (Level 1 or 2) to partial oral intake (e.g., Levels 3 or 4) and eventually to a safer, more independent status (Levels 5 through 7) as therapy unfolds.

**Pediatric Screening–Priority Evaluation Dysphagia (PS-PED)**: The PS-PED [28] is a screening tool specifically developed to identify children who may be at risk for dysphagia or swallowing difficulties. It consists of 14 items that are grouped into three main domains: medical history, health status, and feeding conditions. Each item is scored on a dichotomous (Yes/No) basis, making the tool both straightforward and quick to administer in clinical or community settings. A cumulative score ranging from 0 to 6 suggests a low risk of dysphagia, while a score of 7 to 14 indicates a moderate to high risk that warrants a more in-depth assessment.

**Karaduman Chewing Performance Scale (KCPS)**: The KCPS [29] is a functional assessment tool designed to categorize and quantify a child’s masticatory abilities under real-life conditions. It evaluates the effectiveness of the child’s chewing function and the degree of assistance required by assessing how the child manipulates and processes food boluses in the oral cavity. The scale ranges from 0 (Normal chewing: the child can bite, masticate, and manipulate food without observable difficulties) to 4 (Unable to bite or chew: the child lacks the ability to process solids, necessitating alternative feeding approaches), reflecting different levels of chewing competency and potential needs for therapeutic intervention. For the present investigation the Italian version of the KCPS was used [30].

**Feeding Assessment Scale (FAS)**: FAS [31] is a parent-reported questionnaire specifically designed to capture everyday feeding challenges encountered by children. Rather than relying solely on clinical observation, the FAS taps into the caregiver’s perspective, providing a real-life account of behaviors, difficulties, and successes related to the child’s mealtime experiences. Each item on the questionnaire is rated by frequency, ranging from 0% (“Never”) to 95% (“Always”), with intermediate options like 25% (“A little”), 50% (“Sometimes”), and 75% (“A lot”).

### 2.5. Data Analysis

Sociodemographic and clinical characteristics, along with PS-PED scores, were analyzed using frequency distributions, mean values (±SD), and medians. In addition, the ASHA NOMS, KCPS, and FAS measures were administered at three distinct time points: T0 (pre-intervention), T1 (post-intervention), and T2 (6-month follow-up). Because our data encompass repeated measures from the same participants over time, we employed Friedman’s test to detect overall differences in scores across the three time points under the null hypothesis that the distributions remain constant. Friedman’s test is a nonparametric alternative to repeated-measures ANOVA, particularly suitable here for handling potentially small sample sizes or non-normally distributed data. To quantify the magnitude of any detected differences, we calculated Kendall’s W, an effect size measure reflecting the degree of concordance among repeated measurements. Following Cohen’s guidelines, we interpreted Kendall’s W as 0.1–<0.3 (small effect), 0.3–0.5 (moderate effect), and >0.5 (large effect) [32]. Where Friedman’s test yielded a significant result, we performed post hoc pairwise comparisons using the Wilcoxon signed-rank test to identify which specific time points (e.g., T0 vs. T1, T1 vs. T2) accounted for the observed differences. The threshold for statistical significance was set at α < 0.05. This multi-step approach enabled us to robustly determine not only whether changes occurred but also the magnitude and specific intervals in which significant improvements or declines in feeding-related scores took place.

## 3. Results

Seven children with CS (3 females and 4 males) were recruited for this study, aged between 2 and 12 years old with an mean age of 7.21 (SD = 4.89). In Table 2, the main characteristics are synthetized.

The use of the PS-PED revealed a mean (SD) score of 9.14 (0.69), suggesting a high risk of dysphagia in the analyzed sample. To better understand which items mostly contribute to PS-PED, Table 3 synthetized the frequency and percentage for each item

Concerning feeding abilities, as measured with ASHA-NOMS the Friedman test demonstrated statistically significant differences with a *p* < 0.01 with a large effect size (Kendall’s W value 0.67); the Wilcoxon signed-rank test and the ASHA-NOMS showed statistically significant differences (*p* < 0.05) in scoring at different timings of administration. In particular, ASHA NOMS revealed a significant difference between T0 and T1 (*p* = 0.02) and T0 and T2 (*p* = 0.03), while no differences were found between T1 and T2. Results are synthesized in Table 4.

Concerning chewing abilities, as measured with KCPS, the Friedman test demonstrated statistically significant differences with a *p* = 0.03 with a medium effect size (Kendall’s W value 0.47); the Wilcoxon signed-rank test and the KCPS showed statistically significant differences (*p* = 0.04) in scoring between T0 and T2. Results are synthetized in Table 5.

For what concern feeding and swallowing difficulties during mealtime, as measured with FAS, the Friedman test demonstrated statistically significant differences with a *p* < 0.01 with a large effect size (Kendall’s W value 0.81); the Wilcoxon signed-rank test and the FAS showed statistically significant differences (*p* < 0.05) in scoring at different timings of administration. In particular, FAS revealed a significant difference between T0 and T1 (*p* = 0.02) and T0 and T2 (*p* = 0.02), while no differences were found between T1 and T2. Results are synthetized in Table 6.

## 4. Discussion

The literature on CS underscores how multiple neurological and anatomical anomalies can influence feeding and swallowing. Such complexity necessitates a multidisciplinary approach, where medical and rehabilitative specialists work collaboratively. In this study, we focused on the potential benefits of GIFT, a program targeting a range of factors integral to oral feeding performance (e.g., oromotor skills, postural stability, behavioral strategies, and caregiver education). Our findings align with the premise that a concentrated, individualized therapy model can improve not only swallowing function and chewing ability but also family perceptions of mealtime experiences.

A recent review [33] revealed that evidence to support purely oral–motor, sensory, or pharmaceutical techniques remains limited and of insufficient strength to draw firm conclusions about efficacy. Behavioral interventions—particularly those based on principles of operant conditioning, systematic desensitization, and reinforcement—exhibited methodological shortcomings, they showed preliminary support for helping improve functional feeding outcomes, such as increased oral intake and reduction in disruptive mealtime behaviors.

Despite principles of GIFT, as motor learning and neural plasticity, are in commons with other approaches [34,35], GIFT aligns with recommendations by Umay et al. [36], emphasizing thorough evaluation, individualized treatment plans and the use of oral-motor exercises (where indicated), sensory strategies, and behavioral techniques to foster feeding acceptance; as recommended GIFT also incorporates compensatory measures such as careful positioning, adaptive utensils, and systematic caregiver education to support progress beyond clinical settings [36].

### 4.1. Dysphagia in CS

The use of the PS-PED indicated that the risk of dysphagia in this population is closely tied to the presence of major and minor diagnostic criteria for CS. This finding aligns with the scientific literature [2,5,8], which notes that these children present a high risk of dysphagia, as evidenced by an average score of 9.14 out of 14. Each PS-PED item was examined, enabling the establishment of direct connections between CS diagnostic criteria and dysphagia risk factors—insights that are highly relevant for both clinical and rehabilitative planning.

Consistent with the work of Dobbelsteyn et al. [5], swallowing and feeding difficulties are profoundly influenced by the diagnostic features of CS, particularly cranial nerve dysfunction, which can disrupt the motor and sensory foundations necessary for safe feeding. Notably, the entire sample carried a neurological diagnosis (Item 1), one of the condition’s major criteria, placing all children at an elevated risk of dysphagia.

Structural anomalies affecting the digestive and respiratory systems (Item 4) were detected in 100% of the sample. This finding underscores the significance of anomalies such as choanal atresia and tracheoesophageal fistula, both of which often lead to aspiration and significant bolus-management challenges. A large proportion of children (71.43%, Item 5) also required a tracheostomy and experienced recurrent respiratory infections (71.43%, Item 8), highlighting the need for specialized rehabilitative interventions that address the unique respiratory–swallowing complexities. Although a tracheal cannula is pivotal for supporting breathing, it may influence secretion control, thereby impacting respiratory and swallowing functions during meals [1,5]. Indeed, 85.71% of the sample use a suction machine (Item 9), underlining the need for continuous support to maintain feeding safety.

Items 12 and 13 reveal that 85.71% of these children depend on enteral nutrition, and all of them (100%) consume foods inconsistent with their developmental stage. While these practices serve to reduce aspiration risk and guarantee the necessary nutritional intake, they limit opportunities for sensory exploration and motor development, thus restricting orofacial muscle stimulation and progress in swallowing efficiency [2,3]. These findings emphasize the importance of intensive, incremental food exposure—both for children receiving tube feeding and for those with delayed feeding skills—to foster the sensory and motor components vital for functional swallowing.

Another key consideration is prolonged mealtime duration (over 50 min in 85.71% of cases, Item 14). This issue reflects not only difficulties in swallowing but also behavioral aversions and food refusals. Such behavioral factors are common in CS, as reported in the literature, further complicating the nutritional management of these patients [22].

Overall, the PS-PED has proven particularly effective and predictive for identifying dysphagia risk in children with CS. By offering a comprehensive perspective on the interplay between major/minor diagnostic criteria and swallowing challenges, it emerges as an invaluable tool. Notably, 80% of the PS-PED items relate to features intrinsic to CS, and the screening instrument itself is based on an extensive review of literature from the past decade, with the goal of elucidating correlations between pediatric dysphagia and a range of health conditions [28].

### 4.2. Feeding Abilities in CS

The ASHA NOMS served as a key instrument for assessing swallowing function and oral feeding levels in children with CS. The results demonstrated statistically significant gains in feeding skills among participants who received GIFT. Prior to intervention (T0), most children in the study population fell within the lower ASHA NOMS tiers (Levels 1 and 2). Such low levels indicate major constraints on oral intake, often restricted to smooth, homogeneous textures, and a substantial dependency on non-oral (artificial) nutrition. Indeed, six of the seven participants partially or fully relied on gastrostomy or nasogastric feeding to meet their nutritional requirements, reflecting the serious swallowing and oromotor challenges commonly associated with CS.

Following the GIFT (T1), significant changes were observed. Children demonstrated an overall improvement in feeding level—some progressed from near-total reliance on tube feedings to being able to handle more varied food consistencies safely. This shift underscores the effectiveness of GIFT’s integrative approach, which combines sensory desensitization, oromotor training, and family-centered strategies to promote consistent skill development [37]. By addressing both the physiological (e.g., bolus formation, tongue lateralization) and behavioral (e.g., food aversion, mealtime compliance) aspects of feeding, GIFT enabled participants to broaden their dietary repertoire and reduce the likelihood of aspiration.

Crucially, these gains were sustained over time, as evidenced by the T2 (6-month follow-up) data. Rather than regressing to pre-treatment feeding levels, most children maintained or even further consolidated their swallowing abilities, reflecting successful generalization of therapy skills into everyday life. Parents, having been actively involved in the treatment sessions, played a pivotal role in reinforcing the techniques acquired, which likely contributed to longer-lasting improvements. This is in line with Tessa and colleagues study [38] who found that specially trained parents successfully continued pediatric eating disorder treatment at home and maintained treatment gains. Notably, enhanced swallowing safety and increased acceptance of varied food textures also facilitated the weaning process for some children who had previously depended on a percutaneous endoscopic gastrostomy (PEG). This progression suggests that intensive interventions, when consistently applied and supported at home—especially with the supervision of a SLP [39]—can effectively reduce reliance on artificial nutrition for children with complex clinical profiles like CS.

### 4.3. Chewing Abilities

Children with CS exhibit significant chewing deficits attributable to anatomical anomalies (e.g., orofacial malformations) and neurological impairments affecting cranial nerve function [3,27]. Such anomalies often compromise orofacial muscle coordination and delay the acquisition of fundamental oral motor skills, particularly under the added influence of clinical comorbidities and food refusal behaviors.

At baseline (T0), all participants scored at KCPS Level 4, reflecting severe masticatory impairment—in other words, the children did not engage in effective biting or chewing. However, between the pre-treatment assessment and subsequent evaluations at T1 and T2, there was a statistically significant improvement, indicating the acquisition of more functional chewing skills. Although further advancements remain necessary, these findings highlight the impact of targeted speech therapy and structured feeding interventions in fostering oromotor development.

The observed progress can be understood in light of neuroplasticity and motor learning principles [14]. By repeatedly exposing children to guided exercises and corrective feedback, therapy sessions promoted reorganization of neural pathways responsible for chewing. Crucially, this progress was maintained at follow-up, largely because parents actively reinforced therapy techniques within the home environment. Their ongoing support expanded the opportunities for skill practice across multiple daily meals, thereby generalizing the motor patterns established during therapy to ordinary, everyday contexts. This continuity of practice not only strengthened newly learned oromotor capabilities but also solidified the children’s confidence in handling more challenging food textures, paving the way for further gains in chewing efficiency.

### 4.4. Parents Perspective on Feeding

In addition to clinical evaluations, this study incorporated a parent/caregiver questionnaire to gauge how feeding and swallowing difficulties manifest in the child’s everyday life. The FAS served as a key instrument for identifying the types of food or textures the child rejects, as well as the level of difficulty caregivers encounter at mealtimes. At the initial assessment (T0), the mean FAS score was notably high, underscoring the impact that feeding challenges have on these families’ routines. By the completion of the intensive therapy (T1), caregivers reported a significant decrease in mealtime difficulties, as reflected by a lowered FAS score and confirmed by a *p*-value of 0.02. This finding suggests that parents swiftly perceived improvements in their child’s feeding abilities, highlighting GIFT’s immediate effectiveness in daily contexts.

At follow-up (T2), the FAS scores remained stable, indicating that the progress observed at T1 persisted over several months. This consistency not only demonstrates the durability of treatment effects but also emphasizes that children and caregivers successfully generalized the new feeding skills to real-world situations.

The parental viewpoint is pivotal for two reasons. First, it offers a holistic measure of how well clinical gains (e.g., improved oral motor skills) translate into fewer mealtime struggles. Second, and perhaps more importantly, it evaluates whether the child’s newly acquired abilities genuinely enhance the family’s quality of life. Observing tangible, sustained changes—even months after therapy concludes—validates that the intensive GIFT approach leads to practical, everyday benefits rather than temporary or clinic-bound improvements.

Moreover, the active role parents play in daily practice underpins these lasting outcomes. By consistently applying the techniques learned during therapy sessions, caregivers reinforce the child’s emerging skills in authentic mealtime settings. Their involvement goes beyond simple observation; it ensures continuous feedback, helps adapt strategies to the child’s evolving needs, and facilitates ongoing progress. This integration of clinical guidance into daily life fosters the long-term consolidation of abilities, thereby confirming the practical effectiveness of the GIFT intervention.

### 4.5. Behavioral Issues

Although we did not employ a dedicated tool to quantitatively measure meal behavior, we observed significant differences between pre- and post-treatment evaluations (T0 vs. T1). Analysis of selected items (e.g., 1, 5, 11, 12) from the FAS showed that mealtime behaviors improved over this period, evidenced by reduced refusal of food, decreased tendencies to avoid chewing or holding food in the mouth, less reliance on adult assistance, and overall shorter meal durations. In line with Cerchiari et al. [10], we believe that enhancing feeding skills, specifically by reducing oral hypersensitivity and improving bolus management, played a key role in minimizing the child’s frustration and curbing dysfunctional behaviors.

Additionally, cognitive–behavioral strategies described in Smith [8] supported the management of negative mealtime behaviors. Children with deficits in sensory processing and oral–motor coordination often struggle to accept new or unfamiliar foods. Accordingly, they must first develop appropriate skills to cope with texture and taste variations, after which they are less prone to frustration and dysregulated behavior. In children with complex medical comorbidities, such behavioral approaches can bolster the broader rehabilitation program designed to improve feeding competencies.

Based on our findings, we propose that improved motor and sensory skills not only enhance a child’s willingness to participate in mealtime activities but also reduce dysfunctional behaviors. By systematically introducing behavioral strategies, therapists and caregivers helped facilitate the acceptance of new foods and textures, ultimately streamlining the child’s dietary experience.

### 4.6. Difference Between CHARGE Syndrome, Autism Spectrum Disorder, and Down Syndrome

Earlier investigations [9] have demonstrated the effectiveness of Global Intensive Feeding Therapy (GIFT) in limited cohorts of children with Autism Spectrum Disorder (ASD) and Down Syndrome (DS), underscoring the versatility of the approach in addressing a range of feeding challenges. This study further corroborates that the same foundational rehabilitation principles can be adapted for children with CHARGE syndrome (CS)—despite CS often presenting more complex oropharyngeal and neurological abnormalities than those typically observed in ASD or DS.

In particular, our cohort displayed a higher incidence of oropharyngeal dysphagia, likely driven by anatomical and functional anomalies (e.g., choanal atresia, cranial nerve dysfunction) that are less frequent or absent in ASD or DS. This difference underlines the need to prioritize swallowing safety in children with CS, not only when introducing new foods but also during masticatory training. For example, the Functional Chewing Training (FuCT) [19] component of GIFT was implemented alongside additional precautions to minimize aspiration risk. Beyond enabling the child to acquire foundational chewing skills, FuCT also fosters muscle activation around the pharyngeal and laryngeal areas, supporting airway protection during swallowing [21].

Given the medical complexity often encountered in CS—ranging from tracheostomies to recurring pulmonary infections—intensive rehabilitative sessions spanning two or more weeks become especially valuable. Such an extended timeline allows parents to become proficient in essential therapeutic techniques and behavioral management strategies relevant to feeding, while also familiarizing themselves with assistive devices that a child with CS may rely on (e.g., postural systems, tracheal cannulas, suction equipment). In earlier GIFT programs for ASD and DS, families typically worked on modulating behavioral or sensory issues related to feeding (e.g., food selectivity, reluctance to accept new textures). In contrast, caregivers of children with CS must master a broader array of tasks, such as timing meal administration around PEG feeds, handling airway management during feeding, and ensuring consistent posture and muscle tone stability.

Overall, while GIFT appears beneficial across these three conditions, the specific therapeutic priorities and implementation strategies can differ markedly. Children with ASD or DS might emphasize sensory desensitization or managing hypotonia, whereas for CS, maintaining airway safety and addressing cranial nerve deficits become paramount concerns. These distinctions accentuate the flexibility of GIFT as a methodology that can be tailored to disease-specific needs, demonstrating how shared rehabilitation principles can yield distinct yet effective pathways to improved feeding and swallowing skills for children with varying etiologies.

### 4.7. Study Limitations and Future Prospects

Although the preliminary findings from this study underscore the effectiveness of GIFT in children with CS, several limitations must be acknowledged. First, the existing body of literature on speech–language interventions for feeding and swallowing disorders in CS remains limited. This relative paucity of research constrained our ability to compare or contextualize our results with prior studies. The condition’s rarity, coupled with its highly heterogeneous clinical presentation, often poses challenges in assembling large and methodologically comparable cohorts.

A second limitation concerns the small sample size (*n* = 7). Although the outcomes are promising, having a limited number of participants reduces the generalizability of conclusions to the broader CS population. Moreover, the variability in the type and severity of craniofacial, neurological, and systemic manifestations among these seven children highlights the complex clinical picture typical of CS. Future investigations should increase sample sizes to capture a more diverse range of clinical manifestations, thereby enabling researchers to pinpoint which specific anomalies (e.g., choanal atresia, cranial nerve dysfunction) most strongly influence therapeutic outcomes.

We also recognize the lack of a control group as an important methodological shortcoming. Without a non-treated or differently treated comparison cohort, it is not possible to definitively rule out other factors—such as natural developmental progression or concurrent medical interventions—that might have contributed to the observed improvements. Despite this limitation, the significant gains noted post-intervention reasonably suggest that GIFT was instrumental in driving progress, given the historically severe feeding challenges in CS.

Looking ahead, we propose several avenues for future research. Expanding the study to include a larger number of participants would yield more robust and statistically powerful data. Introducing a control or waitlist group could further clarify the effectiveness of GIFT by contrasting the outcomes of children who receive immediate therapy with those who wait. Such a design might also elucidate the natural trajectory of feeding skill development in CS if left unmanaged or only partially managed through standard medical care. Additionally, incorporating PS-PED more extensively in screening and follow-up protocols could help clinicians better quantify dysphagia risk and monitor how specific risk factors evolve over time or respond to therapy.

Despite these constraints, our experience suggests that early interdisciplinary assessment of feeding and swallowing abilities—combined with a structured, speech–language rehabilitation approach that addresses both management and active rehabilitation—can yield meaningful improvements in children with CS. As data accumulate, evidence-based guidelines for speech and feeding interventions will become clearer, ultimately enabling more targeted, individualized therapies for this complex group.

## 5. Conclusions

The GIFT program demonstrated tangible improvements in swallowing safety, chewing skills, and mealtime behavior in children with CS. Families also reported notable reductions in daily feeding difficulties and expressed a positive, immediate impression of the treatment’s impact. These results establish GIFT as a valuable rehabilitative option for children and families confronting significant feeding and swallowing challenges, while also highlighting the pressing need for continued research with larger, controlled study designs to refine best practices in CS care.

## Figures and Tables

**Table 1:** Diagnostic criteria for CHARGE syndrome.

Major Criteria
Ocular coloboma
Choanal atresia/stenosis
Cranial nerve anomalies
Characteristic ear anomalies
**Minor Criteria**
Cardiovascular malformations
Genital hypoplasia
Cleft lip/palate tracheoesophageal fistula
Distinctive CHARGE facies
Growth deficiency
Developmental delay

**Table 2:** Sample characteristics.

	Child 1	Child 2	Child 3	Child 4	Child 5	Child 6	Child 7	Total
Age (year)	2.11	3.5	5.1	6.6	6.7	10.2	14.9	7.21 (4.89)
Gender	F	F	M	M	F	M	M	M 4 (57.14)
Major criteria								
Coloboma	+	+	+	+	−	+	+	6 (85.71)
Cranial nerve dysfunction	+	+	+	+	+	+	+	7 (100)
Ear anomalies	+	+	+	+	+	+	+	7 (100)
Atresia/Stenosis	−	−	−	−	+	−	−	1 (14.29)
Minor criteria								
Cardiovascular malformations	+	−	−	−	+	+	−	3 (42.86)
Genital abnormalities	−	−	+	−	−	−	−	1 (14.29)
Orofacial clefts	+	−	−	+	+	−	−	3 (42.86)
Tracheoesophageal fistula	−	−	+	−	−	−	−	1 (14.29)
Craniofacial dysmorphisms	+	+	+	+	+	+	+	7 (100)
Growth Deficiency	+	+	+	+	+	+	+	7 (100)
Developmental delay	+	+	+	+	+	+	+	7 (100)
PS-PED	10	9	9	9	10	9	8	9.14 (0.69)
KCPS	4	4	4	4	4	4	4	4
ASHA-NOMS	1	2	4	2	1	3	4	2.43 (1.72)

+: presence of criterium; −: absence of criterium.

**Table 3:** PS-PED results.

PS-PED		
Item	Yes *n* (%)	No *n* (%)
Neurological diagnosis	7 (100)	0 (0.00)
Epilepsy medications	1 (14.29)	6 (85.71)
Heart disease	3 (42.86)	4 (57.14)
Structural anomalies of the digestive and respiratory systems	7 (100)	0 (0.00)
Tracheal tube	5 (71.43)	2 (28.57)
Decreased alertness	0 (0.00)	7 (100)
Malnutrition and/or poor growth	1 (14.29)	6 (85.71)
Recurrent respiratory tract infections	5 (71.43)	2 (28.57)
Use of the suction machine/aspirator	6 (85.71)	1 (14.29)
Lack of head control and/or postural instability	3 (42.86)	4 (57.14)
Gastrointestinal disease (gag reflex, vomit, constipation, GERD)	7 (100)	0 (0.00)
Parenteral/enteral nutrition (nasogastric tube, gastrostomy tube, etc.)	6 (85.71)	1 (14.29)
Feeding with consistency and unsuitable food for the child’s development stage	7 (100)	0 (0.00)
Prolonged mealtime (over 50 min)	6 (85.71)	1 (14.29)

**Table 4:** Difference in scoring of the ASHA-NOMS.

Mean (SD)	T0	T1	Median (IQR)	T0	T1	SIG
	2.43 (1.72)	3.14 (1.46)		2 (1–4)	4 (2–5)	0.02 *
	T1	T2		T1	T2	
	3.14 (1.46)	3.43 (1.62)		4 (2–5)	5 (3–5)	0.16
	T0	T2		T0	T2	
	2.43 (1.72)	3.43 (1.62)		2 (1–4)	5 (3–5)	0.03 *

* *p* < 0.05.

**Table 5:** Difference in scoring of the KCPS.

Mean (SD)	T0	T1	Median (IQR)	T0	T1	SIG
	4.00 (0.00)	3.57 (0.53)		4 (4–4)	4 (3–4)	0.08
	T1	T2		T1	T2	
	3.57 (0.53)	3.29 (0.75)		4 (3–4)	3 (3–4)	0.16
	T0	T2		T0	T2	
	4.00 (0.00)	3.29 (0.75)		4 (4–4)	3 (3–4)	0.04 *

* *p* < 0.05.

**Table 6:** Difference in scoring of the FAS.

Mean (SD)	T0	T1	Median (IQR)	T0	T1	SIG
	69.43 (14.99)	61.57 (18.33)		69 (59–73)	54 (50–68)	0.02 *
	T1	T2		T1	T2	
	61.57 (18.33)	54.00 (21.41)		54 (50–68)	47 (40–58)	0.06
	T0	T2		T0	T2	
	69.43 (14.99)	54.00 (21.41)		69 (59–73)	47 (40–58)	0.02 *

* *p* < 0.05.

## Data Availability

The original contributions presented in this study are included in the article. Further inquiries can be directed to the corresponding author.
